# The Role of Rac GTPase in Dendritic Spine Morphogenesis and Memory

**DOI:** 10.3389/fnsyn.2020.00012

**Published:** 2020-04-17

**Authors:** Joana Freitas Costa, Monica Dines, Raphael Lamprecht

**Affiliations:** Sagol Department of Neurobiology, Faculty of Natural Sciences, University of Haifa, Haifa, Israel

**Keywords:** dendritic spines, Rac1 GTPase, actin cytoskeleton, memory consolidation, memory forgetting, memory extinction, memory erasure

## Abstract

The ability to form memories in the brain is needed for daily functions, and its impairment is associated with human mental disorders. Evidence indicates that long-term memory (LTM)-related processes such as its consolidation, extinction and forgetting involve changes of synaptic efficacy produced by alterations in neural transmission and morphology. Modulation of the morphology and number of dendritic spines has been proposed to contribute to changes in neuronal transmission mediating such LTM-related processes. Rac GTPase activity is regulated by synaptic activation and it can affect spine morphology by controlling actin-regulatory proteins. Recent evidence shows that changes in Rac GTPase activity affect memory consolidation, extinction, erasure and forgetting and can affect spine morphology in brain areas that mediate these behaviors. Altered Rac GTPase activity is associated with abnormal spine morphology and brain disorders. By affecting Rac GTPase activity we can further understand the roles of spine morphogenesis in memory. Moreover, manipulation of Rac GTPase activity may serve as a therapeutic tool for the treatment of memory-related brain diseases.

## Dendritic Spines and Their Role in Memory

Much evidence show that memories are created by alterations in synaptic transmission. These synaptic modifications can be formed by structural changes at postsynaptic sites that can be then actively stabilized over hours or days. These alterations are suggested to form a new neuronal circuit that constitutes the memory trace that, upon memory retrieval, will lead to new intrinsic and possibly behavioral responses. It has been shown that learning leads to structural and functional changes of dendritic spines and that such changes are correlated with memory strength and its retention (Lamprecht and LeDoux, 2004; Caroni et al., 2012; Bailey et al., 2015; Basu and Lamprecht, 2018). Moreover, disruption of such structural changes is associated with memory impairment.

### Structure and Function of Dendritic Spines

Dendritic spines are neuronal dendritic protrusions that contain mainly excitatory synapses (Nimchinsky et al., 2002; Lamprecht and LeDoux, 2004; Newpher and Ehlers, 2009; Nishiyama and Yasuda, 2015). Dendritic spines are subdivided into several categories according to their morphology (Peters and Kaiserman-Abramof, 1970) that include spines with no visible neck (stubby spines), thin spines with a discernable neck and a small head, mushroom spines and branched spines. Mushroom spines are easily distinguished by their short length neck and large head and branched spines have multiple heads that emerge from a common origin (Harris et al., 1992). Interestingly, Tønnesen et al. (2014) showed, using stimulated emission depletion (STED) microscopy, that stubby spines seem to be overreported since short-necked spines may appear to have a stubby appearance in light microscopy (Tønnesen et al., 2014). Dendritic filopodia are protrusions that are involved in sampling for presynaptic partners and are considered as precursors of dendritic spines (Hering and Sheng, 2001; Matus, 2005). As mentioned above, dendritic spines are known to receive mainly excitatory synaptic inputs. However, in recent years, it has been shown that dendritic spines can contain inhibitory synapses and become dually innervated by excitatory and inhibitory inputs (Villa et al., 2016).

Dendritic spines include a dense structure called the postsynaptic density (PSD), which contains necessary components for synaptic transmission such as α-amino-3-hydroxy-5-methyl-4-isoxazolepropionic acid (AMPA) receptors and *N*-methyl-D-aspartate (NMDA) receptors, PSD organization proteins (e.g. scaffolding proteins), and proteins involved in formation and modulation of synaptic structure and transmission (e.g. Eph and ephrins and neurotrophic receptors) and adhesion (Verpelli et al., 2014). In addition to these proteins, spines also contain actin and actin-binding and regulatory proteins that affect spine morphology (Hotulainen and Hoogenraad, 2010; Chazeau et al., 2014; Verpelli et al., 2014). These proteins include F-actin regulatory proteins that are involved in actin nucleation such as formins, WAVE and Arp2/3, proteins that regulate actin polymerization such as cofilin and profilin that are involved in actin depolymerization and polymerization, respectively, and F-actin capping proteins that block the exchange of actin subunits at F-actin barbed end. These actin regulatory proteins are controlled by upstream molecules such as small GTPases that are in turn regulated by guanine nucleotide exchange factors (GEFs) and GTPase-accelerating proteins (GAPs) (Duman et al., 2015). Small GTPase GEFs and GAPs are responsive to synaptic stimulation and thus can mediate between synaptic activation, changes in actin dynamics and structure and spine morphogenesis.

Changes in the morphology of spines may influence neuronal functions that subserve the formation of memory, such as synaptic transmission and efficacy. For example, synaptic transmission is correlated with the dimension of spine structure. It is shown that a higher level of AMPA receptors tends to be found in spines with large postsynaptic densities (PSDs) than in spines with smaller PSDs (e.g. Takumi et al., 1999). Since the dimensions of the spine head are correlated with the area of the PSD (Harris and Stevens, 1989), it is implied that more glutamate receptors are expressed in the spine with larger head than that with smaller head. In addition, a correlation between the spine head volume and the amplitudes of currents in the spine is detected, showing that spine head volume is approximately proportional to the distribution of functional AMPA receptors (Noguchi et al., 2011).

Synaptic efficacy is also affected by the geometry of the spine neck. Changes in the morphology of spine neck appears to affect the amplification of local voltage in the spine and the compartmentalization of biochemical components, such as of Ca^2+^, within the spine head (Noguchi et al., 2005) and affect bidirectional diffusion of material from dendrite to spines and signal transduction (Bloodgood and Sabatini, 2005; Gray et al., 2006; Santamaria et al., 2006). Spines with thinner longer necks confine more molecules. Thus, synaptic efficacy and also neuronal function may be affected by changes in the spine neck (Araya et al., 2006, 2014). For example, small somatic voltage contributions are detected in spines with a long neck. The pairing of synaptic stimulation with postsynaptic activity can lead to the shortening of the spine neck, alterations in the input/output gain and increase in synaptic efficacy in pyramidal neurons (Araya et al., 2014). Tønnesen et al. (2014) show that the spine neck becomes wider and shorter after long-term potentiation. They predict that such morphological changes will preserve overall biochemical compartmentalization and lead to a drop in spine head excitatory postsynaptic potential (EPSP).

The aforementioned observations show that dendritic spine morphology and the content and the activity of molecules within the spine can affect synaptic efficacy and neuronal transmission. Spine morphology and molecular function can be regulated by synaptic activity that is similar to that detected during learning. In turn, changes in these spine parameters that affect neuronal function, such as alteration in the response of the neuron to incoming inputs, may constitute the memory trace. Such a memory trace is expected to last and to subserve long-term memory (LTM) persistence. Indeed, studies indicate that spine structure can last for a long period of time and thus may support an enduring memory trace (Basu and Lamprecht, 2018).

### Dendritic Spines in Memory Formation

Several lines of evidence have shown that changes in dendritic spine morphology and density and modifications in spine PSD are associated with memory formation (Lamprecht and LeDoux, 2004; Bailey et al., 2015). For example, the density of dendritic spines in the anterior cingulate cortex and hippocampal cornu ammonis 1 (CA1) is increased following contextual fear conditioning (Restivo et al., 2009; Vetere et al., 2011). Auditory fear conditioning extinction leads to an increased spine formation in pyramidal neurons located in layer V in the mouse frontal association cortex, whereas fear conditioning induces spine elimination in this brain region (Lai C. S. et al., 2012). Fear conditioning decreases spine head volume in the lateral amygdala (LA) and leads to an increase in the PSD area in the smooth endoplasmic reticulum (sER)-free spines in LA (Ostroff et al., 2010). Auditory fear conditioning leads to putative LA–auditory cortex (ACx) synaptic pairs, as it increases the pathway-specific formation of LA axons buttons in ACx and dendritic spines of pyramidal cells in layer V of ACx (Yang et al., 2016). Animals trained with conditioned place preference show increased levels of spine density within the basolateral amygdala complex (Young et al., 2014). Changes in spines are formed in the motor cortex by learning and is therefore suggested to provide a structural basis for spatial coding of motor memory (Fu et al., 2012). In addition, evidence indicates that spines morphological changes and new spines induced by learning can become stable for a long period of time to maintain LTM. For example, it was shown that the amount of the stable spines is correlated with performance after learning (Yang et al., 2009). Training for a forelimb reaching task leads to new dendritic spine growth that is preferentially stabilized by subsequent training sessions (Xu et al., 2009).

These results show that learning leads to changes in spine morphology and density and that some of the changes in spines persist over a long period of time. However, several questions remain to be clarified. Does spine morphogenesis underlie memory formation and maintenance? What are the molecular mechanisms that mediate such changes and are they involved in memory? Can we affect spine morphology to treat brain diseases? The study of Rac GTPase allows an insight into the role of spine morphogenesis in memory and may provide critical answers for these questions.

## Rac GTPase and Spine Morphogenesis

### Rac GTPase and Its Regulation

Rac is a small (∼21 kDa) GTPase and is a member of the Rho family of GTPases. Rac GTPase family consists of four members Rac1–3 and RhoG. Rac GTPase cycles between GTP (active)-bound state and GDP (inactive)-bound state (Heasman and Ridley, 2008). The GTP/GDP cycle is regulated by proteins that activate Rac GTPases by catalyzing GDP/GTP exchange (GEFs) or inhibit Rac GTPases by stimulating GTP hydrolysis (GAPs) (Duman et al., 2015).

**FIGURE 1:**
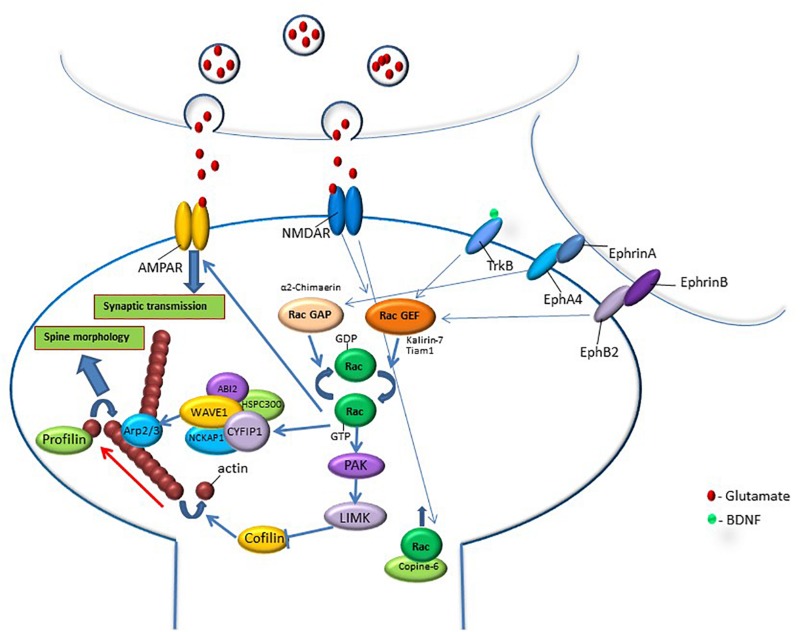
Rac GTPase activity is regulated by synaptic activation through synaptic receptors that are known to be involved in memory formation such as NMDA, Trk, and Eph receptors. Activation of these synaptic receptors leads to the regulation of RacGEFs or RacGAPs that activate or inhibit Rac GTPase, respectively. Rac GTPase regulates downstream effectors that can affect spine morphology and synaptic transmission. Rac GTPase exerts its effects on spine morphology through the regulation of molecules that affect signaling pathways that control actin-regulatory proteins. In its active state, Rac GTPase activates the PAK-LIMK-cofilin pathway that can control actin dynamics through the inhibition of cofilin, an actin-depolymerizing protein. Rac GTPase also regulates actin network through the modulation of the WAVE-Arp2/3 pathway that, in turn, regulates actin nucleation and actin branching. Both cofilin and Arp2/3 regulation of the actin cytoskeleton affect spine morphology. In addition, Rac GTPase may regulate synaptic efficacy by controlling AMPA receptor content in the synapse. Rac1 GTPase protein level in the spine and its effects on spine functions can be regulated by calcium-induced translocation of Rac1 GTPase into the spine by copine-6. The correlation between Rac GTPase activity and changes in spine morphology, synaptic transmission and memory indicates that Rac GTPase regulates spine functions that can mediate various stages of memory formation, erasure, extinction and forgetting.

Rac1 GTPase activity is regulated by synaptic extracellular signaling through synaptic receptors involved in spine morphogenesis (Penzes et al., 2008; Figure 1). For example, it has been shown that NMDA receptor-induced CaMKII activation is important for spine plasticity (Maletic-Savatic et al., 1999; Jourdain et al., 2003; Matsuzaki et al., 2004). NMDA receptor activation induces phosphorylation of the GEF kalirin-7, in a CaMKII-dependent manner, leading to the activation of small GTPase Rac1 and rapid enlargement of spines. Moreover, it is shown that kalirin is required for the long-term maintenance of spines (Xie et al., 2007). Additional study has shown a role for kalirin-7 regulator disrupted-in-schizophrenia 1 (DISC1) and Rac1 in modulating the structure and function of spines (Hayashi-Takagi et al., 2010). Another example of a Rac-GEF that is responsive to glutamate stimulation is Tiam1 that can be phosphorylated by CaMKII (Fleming et al., 1999). NMDA-receptor-mediated increase in calcium results in phosphorylation of Tiam1 and the increase in its GEF activation (Tolias et al., 2005). Tiam1 knockdown was shown to reduce dendritic spine density and lead to the simplification of neuronal dendritic tree (Tolias et al., 2005). Tiam1 is also activated by brain-derived neurotrophic factor (BDNF) and its receptor tropomyosin-related kinase B (TrkB). TrkB phosphorylation at S478 controls its interaction with Tiam1 leading to Rac1 activation during activity-dependent dendritic spine remodeling (Lai K. O. et al., 2012). In neurons that express a dominant-negative form of Tiam1, the induction of spine formation and enlargement by BDNF was abolished (Lai K. O. et al., 2012). Another study has shown that the spreading of Rac1 signaling out of the stimulated spine, mediated by BDNF–TrkB activation, is needed for facilitating structural long-term potentiation (sLTP) in nearby spines (Hedrick et al., 2016). Rac1 GTPase is also regulated by additional synaptic receptors such as EphB, whose activation leads to Rho-GEF kalirin translocation to the synapse and activation of Rac1 and its downstream effector p21-activated kinase (PAK). Overexpression of dominant-negative EphB receptor eliminates ephrin-induced spine development (Penzes et al., 2003). EphA-mediated spine morphogenesis in hippocampal neurons is suppressed by disruption of the Rac-GAP α2-chimaerin (Iwata et al., 2015). Synaptic signaling that activates PKC is also involved in Rac1-mediated synaptic morphogenesis, as it was shown that molecular pathways that involve Rac1 and Rho-A and are activated by PKC produce actin-based structural plasticity in dendrites and spines (Pilpel and Segal, 2004).

Thus, Rac GTPase is regulated by synaptic receptors and signaling molecules that have been shown to be involved in memory formation, such as NMDAR, EphB, and TrkB. Therefore, Rac GTPase can mediate between synaptic activation during learning and cellular and molecular activities that affect changes in spine morphology and synaptic transmission that underlie synaptic plasticity and memory formation. The actin cytoskeleton subserves neuronal morphology and synaptic transmission, and its dynamics and structure are intimately mediated by synaptic activation and Rac GTPase activity. Thus, the actin cytoskeleton appears to mediate between learning-induced Rac GTPase activity and cellular events that mediate memory formation.

### Rac GTPase-Regulated Pathways and Their Effects on the Neuronal Actin Cytoskeleton

The active Rac GTPase exerts its effects by binding and activating different effectors. One such example is the activation of PAK by Rac. Active PAK, in turn, phosphorylates LIM kinase (LIMK), which will then phosphorylate cofilin inhibiting, consequently, its actin depolymerization activity affecting, therefore, the content of actin filaments (Lappalainen and Drubin, 1997; Arber et al., 1998; Yang et al., 1998; Edwards et al., 1999). This pathway affects the actin cytoskeleton network in the dendritic spine and spine morphology. For example, the normal distribution of filamentous actin (higher level of filamentous actin in spines compared to the adjacent dendritic area) in hippocampal neurons, was disrupted in LIMK-1 knockout (KO) neurons. These KO neurons show a low level of actin filaments in spines, which is not significantly higher than that of other dendritic areas, as opposed to neurons from wild-type animals showing higher filamentous actin in spine heads compared to the adjacent dendritic area. These results indicate that the high level of actin filaments in spines is maintained by LIMK activity (Meng et al., 2002). LIMK regulates actin polymerization by phosphorylation and inhibition of cofilin (Arber et al., 1998). Cofilin regulates actin dynamics and spine morphology. Mice in which n-cofilin (a non-muscle cofilin) was removed postnatally (N-cofflx/flx,CaMKII-cre) from principal neurons exhibit an increase of 50–60% in the F/G-actin ratio in hippocampus and cortex synaptosomes when compared to controls (Rust et al., 2010). Decreased amounts of cofilin-1 lead to a decrease in actin filament turnover rates in spines (Hotulainen et al., 2009).

The aforementioned observations show that molecules in this Rac1-cofilin pathway (Rac, PAK, LIMK, and cofilin), as well as in other pathways (e.g. Rac1-WAVE, see below), are intimately involved in the regulation of actin cytoskeleton structure. The actin cytoskeleton was shown to mediate the formation and elimination, morphology, motility and stability of dendritic spines (Halpain et al., 1998; Matus, 2000; Schubert and Dotti, 2007; Honkura et al., 2008; Hotulainen and Hoogenraad, 2010; Chazeau et al., 2014). Actin polymerization affects spine head structure. For example, glutamate-stimulation-induced spine head enlargement is dependent on actin polymerization (Matsuzaki et al., 2004). In addition, actin may be involved in affecting the morphology of the spine neck. A biophysical model suggests that the stabilization of spines is assisted by the constriction of the spine neck, thus pointing to a role in stabilization and maintenance for F-actin ring-like structures that are consistently found in the spine neck (Miermans et al., 2017). Thus, Rac GTPase can affect spine morphology through the control of actin-regulatory proteins.

### Rac GTPase Effects on Dendritic Spine Morphogenesis

Modulation of Rac GTPase activity leads to changes in neuronal morphology of dendritic spine. For example, expression of a dominant-negative form of Rac1 results in the elimination of dendritic spines in the hippocampus (Nakayama et al., 2000), and RacV12 (constitutively active mutant) increases spine density and reduces spine size (Tashiro et al., 2000). Spines in Purkinje neurons of transgenic mice expressing constitutively active Rac1 are reduced in size but increased in number (Luo et al., 1996). The mean spine head area is significantly larger, and the mean PSD length is significantly increased in mice that are devoid of Rac1 in excitatory neurons in the forebrain (Haditsch et al., 2009). Overexpression of a dominant-negative Rac1 (Rac1-DN) in cultured primary hippocampal neurons led to a reduced spine density, to longer and thin filopodia-like spine, and reduced synaptic motility. On the other hand, more lamellipodia-like synapses with a large spine head are produced following the expression of constitutively active Rac1 (Rac1-CA) (Liu et al., 2016). Activation by light of a photoactivatable Rac1 GTPase (activated synapse targeting photoactivatable Rac1; AS-PA-Rac1) in selected spines of neurons in the motor cortex leads to shrinkage of the AS-PA-Rac1-containing spines (Hayashi-Takagi et al., 2015). Overexpression of Rac1 in hippocampal slices induced a significant increase in spine density and AMPAR clustering, leading to an increase in both frequency and amplitude of miniature excitatory synaptic currents (mEPSCs) (Wiens et al., 2005). Besides Rac1 role in spine morphogenesis, Rac3 isoform also displays a role in spine morphology since overexpression of either Rac1 or Rac3 GTPases leads to an increase in spine density (Pennucci et al., 2019). Rac3 is more effective in promoting mature spine enlargement and Rac1 appears to be more efficient in inducing spine formation. Double knockout of both Rac1 and Rac3 genes inhibits the formation of dendritic spines and induces an increase in dendritic protrusions such as filopodia (Corbetta et al., 2009; Pennucci et al., 2019).

Rac GTPase effectors are also involved in the regulation of spine morphology. PAK is involved in neuronal morphogenesis. Cortical neurons in the forebrain of dominant-negative PAK (dnPAK) transgenic mice exhibit fewer spines and an increase in the proportion of larger synapses (Hayashi et al., 2004). PAK exerts its effects through LIMK and cofilin. LIMK can be directly phosphorylated and activated by PAK (Edwards et al., 1999). In LIMK knockout mice, most of the spines have thick neck and small head in contrast to wild-type mice where the majority of spines have large head and thin neck (Meng et al., 2002). LIMK phosphorylates and inhibits cofilin function (Arber et al., 1998). Active cofilin promotes actin depolymerization and is an essential regulator of actin dynamics in neurons (Rust, 2015). N-cofflx/flx,CaMKII-cre mice (n-cofilin removed postnatally) exhibit enlargement of dendritic spines and an increase in synapse density in the hippocampus (Rust et al., 2010). A decrease in cofilin leads to abnormal spine morphology and neurons typically contain abnormal filopodia-like protrusions or have aberrantly long spine necks (Hotulainen et al., 2009). In addition to LIMK/cofilin effectors, Rac1 affects actin cytoskeleton rearrangements in spine, through the regulation of the WAVE protein that consequently acts upon actin nucleator actin-related protein 2/3 (Arp2/3) to drive Arp2/3 activities, leading to the formation of dendritic spines (Sanchez et al., 2009). Arp2/3-containing complex binds to the side of a preexisting actin filament to form a new branch of actin filament (Pollard, 2007). Arp2/3 is concentrated in dendritic spines and is necessary for activity-dependent spine enlargement for spine head growth (Kim et al., 2006, 2013; Rácz and Weinberg, 2008; Wegner et al., 2008; Hotulainen et al., 2009). Arp2/3 activity was shown to be essential for the formation of LTM (e.g. Basu et al., 2016).

Interestingly, Rac1 activity is involved in the maintenance of spine morphology. Induction of sLTP (structural enlargement of dendritic spine) using uncaging of glutamate on a single spine led to the activation of Rac1 that lasted more than 30 min (Saneyoshi et al., 2019). Inhibition of Rac1 by EHT1864 postinduction blocked sLTP, showing that consistent activation of Rac1 by RacGEF is needed for the maintenance of sLTP. Furthermore, single spine stimulation leads to the rapid formation of a complex consisting of CaMKII and Tiam1 and to constitutive CaMKII activation, which persistently phosphorylates Tiam1. Spine structure is maintained during LTP by phosphorylation of Tiam1 that promotes stable actin-polymerization through Rac1.

### Additional Neuronal Functions Regulated by Rac GTPase

In addition to its role in spine regulation, Rac is also involved in other neuronal functions that may contribute to alterations in synaptic efficacy and changes in neuronal ensembles. For example, Rac is involved in axonal morphogenesis (e.g. Spillane and Gallo, 2014) that may contribute to the formation of new neuronal connectivity that subserves memory. Furthermore, Rac1 is also involved in neurogenesis since a learning-induced increase in neurogenesis in the adult mouse hippocampus is impaired in neurons that are devoid of Rac1 (Haditsch et al., 2013). Neurogenesis is involved in memory formation (Gonçalves et al., 2016). Rac has been associated with the determination of the molecular composition and function at the synapse, such as the regulation of AMPA receptor functions. It was shown that breakpoint cluster region (BCR) protein, a RacGAP (see below), is important for regulating AMPA receptor localization on the neuronal surface, both at basal level and in response to EphB activation (Um et al., 2014). Moreover, Rac1 activation enhances recruiting AMPARs to the synapses during spinogenesis (Wiens et al., 2005). Another recent study shows that, on memory retrieval after long cocaine withdrawal, matured synapses become AMPAR silent again, followed by rematuration ∼6 h later. Increase and decrease in Rac1 activities control these synaptic dynamics, leading to the closing and opening, respectively, of the silent synapse-mediated destabilization window (Wright et al., 2020).

Taken together, the aforementioned results show that Rac GTPase is intimately involved, through its effectors, in regulating spine morphology. The effect of Rac GTPase on spine morphology may depend on the localization of the neurons in the brain and the time of activation. Moreover, different experimental protocols can account for different effects. Since spine morphogenesis is involved in memory formation and Rac GTPase is involved in this process, the observations beg the questions: Is Rac necessary for memory and does its involvement in memory correlates with its effects on spine morphogenesis?

## Rac1 GTPase in Memory Formation, Forgetting, Erasure and Extinction

### Rac1 GTPase in Learning and Memory Formation

The above observations show that Rac GTPase is involved in different cellular events such as changes in neuronal morphology and synaptic transmission that are believed to be necessary for memory formation. Indeed, Rac1 GTPase is shown to be involved in memory formation. Several studies have demonstrated a role for Rac1 GTPase in learning and memory formation in the amygdala. Light stimulation of photoactivatable Rac1 (PA-Rac1) during auditory fear conditioning in the LA led to an increase in PAK phosphorylation and to an impairment in long- but not in short-term auditory fear conditioning memory (Das et al., 2017). Inhibiting the downstream effector PAK in LA enhanced long- but not short-term fear conditioning memory. Rac1 function in astrocytes in the basolateral amygdala (BLA) is also important for long-term fear memory formation. Photoactivation of PA-Rac1 in astrocytes led to their structural alterations, and activation of Rac1 in astrocytes in BLA during fear conditioning attenuated fear memory formation (Liao et al., 2017). In another study, short- and long-term auditory fear conditioning memories are impaired when Rac1 is deleted from excitatory neurons but not from parvalbumin inhibitory neurons. Conditional knockout of Rac1 before fear conditioning training in the BLA impaired short- and long-term fear memories. The expression of a dominant-negative mutant of Rac1 in BLA, or infusion into BLA of Rac1 inhibitor NSC23766 before or after fear conditioning, blocked both fear conditioning short- and long-term memories (Gao et al., 2015). Taken together, the results show that alteration of Rac1 activity in the amygdala during or after fear conditioning affects memory formation.

In addition to memory consolidation, Rac1 is also involved in memory reconsolidation in the BLA. Reconsolidation of auditory Pavlovian fear memory was impaired when NSC23766 was microinjected into the BLA but not into the central amygdala (CeA) or hippocampal CA1 immediately after retrieval of auditory fear conditioning memory (Wu et al., 2014).

Other types of memories such as spatial and contextual memories are also mediated by Rac1 activity. Systemic administration of NSC23766 improved contextual fear memory at 1.5 and 24 h memory tests (Gan et al., 2016). Microinjection of NSC23766 after fear memory retrieval into the hippocampal CA1, but not into CeA or BLA, disrupted contextual fear conditioning memory reconsolidation (Wu et al., 2014).

Rac1-deficient mice (by CaMKII promoter-driven excision of Rac1 using a Cre-lox system) are impaired in learning and memory. More time was required to locate the hidden platform in the Morris water maze task in these Rac1-deficient mice, suggesting that learning is impaired in these mice. In the delayed matching-to-place (DMP) Morris water maze version, a behavior that depends on the integrity of NMDA receptor activation in hippocampus (Steele and Morris, 1999), the escape latency was reduced in a much faster rate in the control mice compared to that in the Rac1-deficient mice, showing that the ability to acquire a memory of the platform location is impaired in the Rac1 mutants (Haditsch et al., 2009). In another study, the authors (Haditsch et al., 2013) show that the selective ablation of Rac1 in postmitotic forebrain projection neurons leads to an impairment in working memory in the DMP task.

Rac1 in nucleus accumbens (NAc) is also involved in cocaine conditioned place preference (CPP) and in cocaine-induced spine morphogenesis (Dietz et al., 2012). Expression of a constitutively active mutant of Rac1 (Rac1-CA) in NAc blocked place preference conditioning and the acute locomotor-activating effect of the drug. Supporting these results, the authors show that activation of PA-Rac1 prevented CPP formation to cocaine. In contrast, intra-NAc injection of a dominant-negative mutant of Rac1 (Rac1-DN) promoted CPP to cocaine without altering locomotor responses. Constitutive active cofilin (cofilin-CA) expression in the NAc also increased the rewarding effects of cocaine. The study further showed that five doses of cocaine led to an increase in the number of dendritic spines on NAc medium spiny neurons, compared with the control mice that were treated with saline when tested 4 h after the last dose. Rac1-CA completely blocked this increase. However, in basal conditions, Rac1-CA had no effect on spine density. Cocaine induction of spines was also blocked by PA-Rac1 when it was activated by light following each injection of cocaine. In contrast, an increase in spine density in saline-treated mice is observed when Rac1-DN is overexpressed. Spine density increase that is induced by cocaine was largely driven by an increase in the number of thin spines. Such an increase was blocked by Rac1-CA and mimicked by Rac1-DN overexpression. The number of mushroom spines is reduced by Rac1-CA but not by Rac1-DN or cofilin-CA.

Rac1 signaling is also crucial for methamphetamine (METH)-induced CPP and structural plasticity (Tu et al., 2019). Expression of Rac1-CA in nucleus accumbens blocked the METH-induced increase in dendritic complexity, length, and branch number and significantly decreased the CPP scores in the group trained for METH CPP. In the saline CPP group, the total spine density is increased by Rac1-DN (mainly by the increased thin spine density), whereas mushroom spine density is significantly increased by Rac1-CA. In the METH CPP group, total spine density was increased and was blocked by Rac1-CA.

The aforementioned results show that the integrity of Rac1 activity is needed for memory formation. Interference, with Rac1 activity, by either activation or inhibition, affects normal memory consolidation (or reconsolidation). Moreover, interference with Rac1 levels of activity also affects neuronal morphology. Thus, precise spatiotemporal Rac1 activity is necessary to form normal memory.

The above studies show that altering Rac1 level or activity directly by pharmacology, by mutating the Rac1 protein or altering Rac1 level by genetic manipulation in the brain, affect LTM formation. Rac1 activity is also modified by controlling Rac GEFs and GAPs. BCR and active BCR-related (ABR) proteins (Heisterkamp et al., 1989) show Rac-GAP activities (Diekmann et al., 1991; Heisterkamp et al., 1993; Tan et al., 1993; Chuang et al., 1995; Voncken et al., 1995; Kaartinen et al., 2001; Cho et al., 2007). Mice deficient of BCR or ABR exhibit enhanced basal Rac1 activity and a small increase in spine density (Oh et al., 2010). The study indicates that the maintenance ability, but not induction, of LTP measured in Schaffer collateral (SC)–CA1 pyramidal neuron synapses of BCR^–/–^ and ABR^–/–^ hippocampus is reduced in mice deficient of BCR or ABR. In contrast, LTD was comparable in wild-type (WT) and knockout (KO) mice. A slight increase in dendritic spine density is observed in the BCR^–/–^ and ABR^–/–^. BCR^–/–^ and ABR^–/–^ mice showed reduced learning relative to WT mice in the Morris water maze. In the probe test, ABR^–/–^ mice, but not BCR^–/–^ mice, spent less time in the target quadrant when compared with WT mice, and in both BCR^–/–^ and ABR^–/–^ mice, the number of exact crossings over the former location of the platform was reduced, suggesting that mice that are deficient in BCR/ABR are impaired in spatial learning and memory (Oh et al., 2010). In the object recognition task, BCR^–/–^ and ABR^–/–^ mice exhibit an equal preference for the two objects, whereas the WT mice show preference to the novel object, suggesting that object recognition memory is impaired in the BCR/ABR-deficient mice. A different Rac GAP protein is encoded by the ArhGAP15 gene (Seoh et al., 2003). It was found that there are fewer CR+, PV+, and SST+ inhibitory neurons in the CA3 and DG regions of the hippocampus of the ArhGAP15^–/–^ mice (Zamboni et al., 2016). The balance between excitatory and inhibitory synapses is altered in primary cultures of hippocampal neurons from the mutant mice, showing overexcitation and reduced synchronicity. Neuritogenesis in primary cultures of dissociated embryonic brains of mutant mice also displayed reduced efficiency of neurite elongation and branching and a simpler neuronal morphology. Anti-ArhGAP15 short hairpin RNA (shRNA) application led to a reduction in spine density compared to controls in hippocampal-cultured neurons. It was also found that hippocampus-dependent working and associative memories are impaired in adult ArhGAP15^–/–^ mice. ArhGAP15^–/–^ mice exhibited normal ability to learn hidden-platform water maze; however, they showed difficulties to locate the new platform position during the reversal phase. ArhGAP15^–/–^ mice also have defects in radial maze acquisition and repetition. Mutant mice were impaired in freezing responses during auditory trace fear-conditioning training and the subsequent tone and context tests subjected 24 h later.

Rac GEFs are also involved in the regulation of spine morphology and memory formation. Kalirin is a brain-specific GEF. In kalirin KO mice, Rac1 activity was shown to be reduced in the cortex but not in the hippocampus (Cahill et al., 2009). Spine density in the KO mice frontal cortex, but not in the hippocampus, is significantly reduced as revealed by Golgi staining of neurons. Two-trial matching to sample in the Morris water maze task was impaired in these mutant KO mice. Moreover, in the five testing days, the KO mice failed to improve their performance in the second vs. the first trial, indicating that their spatial working memory abilities are impaired. The same KO mice also presented severe difficulties in the Y-maze arm recognition task, which indicates an impairment in working memory.

Rac1 localization in neurons can also be regulated by neuronal activity and may affect neuronal morphogenesis, synaptic plasticity and memory. One protein that affects Rac1 localization is copine-6 (Reinhard et al., 2016). Copine-6 binds Rac1 and recruits it to the postsynaptic spine membrane in response to calcium influx. Chemical LTP (cLTP)-triggered calcium transients translocate copine-6 from the dendrite to the membrane of postsynaptic spine. Copine-6 translocation into postsynaptic spines is also triggered by calcium influx via NMDA receptors. Rac1 accumulates in spines following cLTP induction but not in the presence of the calcium mutant Copine-6D167N-myc. cLTP leads to an increase in spine head width but not in neurons of Cpne6 KO mice. Moreover, hippocampal learning and memory and synaptic plasticity require copine-6. Stimulation of Schaffer collaterals in acute hippocampal slices leads to LTP in WT mice, but the increase in the EPSPs responses in Cpne6 KO mice returns to baseline within 60 min. Cpne6 KO mice show impairment in fear-conditioning learning and in context, but not cued, dependent long-term fear memory.

The aforementioned results show that Rac activity is regulated in the brain by upstream GAPs and GEFs and is affected by synaptic activity. Moreover, impairment in Rac1 activity and cellular localization leads to alterations in spine morphology and memory formation. Thus, Rac1 can mediate between synaptic activation (e.g. during learning) and cellular events that underlie memory.

### Rac1 GTPase in Memory Erasure

To understand the role of Rac1 in the regulation of spine morphology and to further elucidate the roles of spine structure in memory, an AS-PA-Rac1 was developed (Hayashi-Takagi et al., 2015). This construct contains a modified photoactivatable Rac1 GTPase that is fused to PSDΔ1.2 and is regulated by dendritic targeting element (DTE) of Arc mRNA. In this manner, NMDA receptor-dependent synaptic activation leads to the target and translation of the construct in activated dendritic segments. Indeed, in mice trained with the rotarod training task, spines were labeled, and the mice exhibited significantly more structural potentiation (enlargement or formation of spines) compared with the non-trained mice in cortical layers II/III of the primary motor cortex (M1). The authors further show that the specific AS-PA-Rac1-containing spines shrink following low-frequency pulsed photoactivation of AS-PA-Rac1. Light activation of M1 immediately after or 1 day, but not 2 days, after training disrupted the acquired learning. The authors suggest that spine potentiation visualized by AS-PA-Rac1 that are evoked by learning (at +1 day), but not spontaneous potentiation (at +2 day), accounts for the memory traces. Thus, the study suggests that shrinkage of potentiated spines by Rac1 GTPase disrupts long-term motor memory in the cortex.

The current results show that Rac1 activity affects learning-acquired task a day after training and that this Rac1 activation leads to shrinkage of the spine. Thus, the results indicate that Rac1 activity should be controlled after training in neurons to maintain the learning-induced neuronal ensembles that mediate the acquired task.

### Rac1 GTPase in Memory Forgetting

Memory is stored in the brain after acquisition and consolidation. Owing to a large number of memory engrams that can be stored over time, it seems reasonable that the brain has a mechanism to remove memories that become unused, also termed as “active forgetting”. Active forgetting may be achieved by the degradation of the cellular and molecular memory traces or the engram cell circuit (Davis and Zhong, 2017). Several studies have shown that Rac1 is involved in memory forgetting. For example, expressing a constitutively active form of Rac1 in the mushroom body neurons (MBn) of *Drosophila* accelerates forgetting of Pavlovian odor-shock olfactory aversive conditioning, whereas expressing a dominant-negative form of Rac1 transgene in MBn inhibits intrinsic forgetting (Shuai et al., 2010). Rac1 downstream effectors are also involved in active forgetting. Memory decay is slowed by constitutively active cofilin, and overexpression of constitutively active Rac1 mutant that is unable to bind PAK fails to accelerate memory decay. The scaffolding protein Scribble forms a signaling complex that includes Rac1, PAK3, and cofilin in the MBn (Cervantes-Sandoval et al., 2016). Scribble knockdown significantly reduces the level of p-cofilin, and when Scribble expression is reduced, memory loss is impaired. Scribble consequently has been pointed out as a mediator of active forgetting in *Drosophila* (Cervantes-Sandoval et al., 2016).

The roles of Rac1 in memory forgetting have also been tested in mice. Toward that end, the investigators expressed Rac1-DN or Rac1-CA in the hippocampus and studied the effects on forgetting of hippocampal-dependent memory (Liu et al., 2016). The inhibition of Rac1 activity in hippocampal neurons, through the targeted expression of a Rac1-DN, extended object recognition memory from less than 72 h to over 72 h, whereas accelerated memory is decayed by Rac1 activation to less than 24 h. In addition, interference-induced forgetting of this memory [where retroactive interference objects were introduced 22 h after sampling (training)] was correlated with Rac1 activation and was blocked by inhibition of Rac1 activity. LTP decay in the Schaffer collateral pathway is also highly regulated by Rac1 activity. Rac1 activation accelerated the decay of LTP, whereas its inhibition slowed LTP decay. Paired pulse low-frequency stimulation (PP-LFS; 900 paired pulses delivered at a frequency of 1 Hz; at 50-ms interval) failed to reverse LTP in the Rac1-DN slices when it was introduced 1 h after the LTP recordings, but significantly induced LTD in the Rac1-CA slices. These patterns are similar to the roles of Rac in memory forgetting. Thus, the aforementioned observations show that activation of Rac1 within excitatory neurons in the hippocampus causes time-based natural decay and interference-induced forgetting of object recognition memory.

A recent study shows that contextual fear conditioning induces Rac1 activation and expression of α2-chimaerin, a RacGAP, in the hippocampus. Furthermore, it is shown that Rac1 activity mediates reversible forgetting. α2-Chimaerin, through inhibition of Rac1 activity during the maintenance stage, reverses forgetting to sustain memory (Lv et al., 2019).

Rac1 is also involved in forgetting of social recognition memory (SRM). A recent study shows that social isolation does not affect SRM (social discrimination paradigm) formation but rather accelerated SRM decay, suggesting enhanced forgetting (Liu et al., 2018). Inhibition of Rac1 activity in both the dorsal and ventral regions of the hippocampus, using Rac1-DN, blocked forgetting of SRM in isolated mice. Activation of Rac1 in the hippocampus, using Rac1-CA, accelerated forgetting in group-housed mice. Accelerated LTP decay in hippocampal slices from isolated mice was rescued by inhibition of Rac1 activity. The observations show that enhanced Rac1-dependent forgetting mediates social memory impairments in isolated mice.

These results further support the observations that controlled Rac1 activity posttraining, in various behavioral paradigms, regulates the strength of memory and that manipulating Rac1 activity affects the maintenance of memory and can lead to memory erasure and forgetting.

### Rac1 GTPase and Memory Extinction

Memory extinction occurs when the conditioned stimulus (CS) cues are subjected alone after learning without the unconditioned stimulus (Pavlov, 1927) leading to relearning of a new association of the CS with the absence of the original reinforcement. Memory extinction does not reflect the forgetting of the original learning (Rescorla, 1996). Rac1 GTPase is also involved in memory extinction. Mass extinction of contextual fear conditioning in rats upregulated Rac1 activity in the hippocampus and led to long-term extinction of contextual fear in rats. Extinction of contextual fear memory is prevented by intrahippocampal injection of the Rac1 inhibitor NSC23766 immediately after extinction trial (Jiang et al., 2016). Long-spaced extinction downregulated Rac1 activity in the hippocampus and led to lesser extinction. Intrahippocampal injection of Rac1 activator CN04-A during extinction trials facilitated the extinction of contextual fear in long-spaced extinction trained rats.

Extinction of conditioned place aversion (CPA) to naloxone-precipitated opiate withdrawal activates Rac1 in the ventromedial prefrontal cortex (vmPFC) of rats. Active Rac1 is needed and sufficient for GABA_A_ receptors (GABA_A_R) endocytosis and CPA extinction (Wang et al., 2017). Knockdown of Rac1 by shRNA within the vmPFC suppressed endocytosis of GABA_A_R and extinction of CPA, whereas expression of a constitutively active Rac1 accelerated GABA_A_R endocytosis and CPA extinction.

## Rac1 GTPase in Brain Disorders

Taking into consideration the central role of Rac1 in memory and neuronal morphogenesis, it is not surprising that this small GTPase has been associated with several brain diseases, that lead to cognitive and psychiatric dysfunctions and neurodegeneration, that involve also abnormalities in neuronal morphology (Newey et al., 2005). Here, we present several examples.

Fragile X Syndrome (FXS) is caused by a reduced or loss of expression of the fragile X mental retardation 1 (FMR1) gene that encodes the Fragile X mental retardation protein (FMRP). FMRP regulates mRNA translation (Bagni et al., 2012; Bhakar et al., 2012). FXS causes impaired cognition, social/language deficits, hyperactivity, hypersensitivity to sensory stimuli, increased susceptibility to seizures, sleep disturbances, attentional deficits and motor incoordination. The neuroanatomical hallmark of FXS is an overabundance of immature dendritic spines in mammals (Rudelli et al., 1985; Irwin et al., 2001). Studying Fmr1 in *Drosophila* revealed that Fmr1 affects dendritic development and that Rac1 is involved in promoting dendritic branching (Lee et al., 2003). Moreover, the study shows that Fmr1 and Rac1 interact genetically with each other in controlling the formation of fine dendritic branches. Another study has shown that in *Drosophila*, the cytoplasmic FMRP-interacting protein (CYFIP) associates Rac-dependent cytoskeleton remodeling and dFMR1-dependent control of translation (Schenck et al., 2003). Studies using Fmr1 KO mice reveal memory impairments (Dutch-Belgian Fragile X Consortium., 1994) and altered spines morphology in these mice (He and Portera-Cailliau, 2013). Rac1 is upregulated in Fmr1 knockout mice (Bongmba et al., 2011). Activation of Rac1 and its effector p21-activated kinase (PAK), by theta burst afferent stimulation (TBS), is impaired at hippocampal synapses in the Fmr1 KO, an FXS mouse model (Chen et al., 2010). Aberrantly increased activity of Rac1 inhibited the actin-depolymerizing factor cofilin and led to spine abnormalities, which are associated with the disease, in the somatosensory cortex of FXS model mice (Pyronneau et al., 2017). Expression of a constitutively active cofilin mutant (cofilinS3A) in the somatosensory cortex of the Fmr1-deficient mice rescued immature dendritic spine and increased spine density phenotypes. Inhibition of PAK1 rescued synaptic signaling and improved sensory processing in FXS mice. Fmr1 KO mice treated with NSC23766, which blocks Rac1 activation by GEFs, exhibited an increase in contextual memory after delayed fear conditioning (Martinez and Tejada-Simon, 2018). Contextual memory is impaired in untreated KO mice compared to that in wild-type mice. In addition, treatment of Fmr1 KO with a Rac1 inhibitor improves cue memory in mice trained for trace fear conditioning training. Treatment with NSC23766 also increases LTP in the Fmr1 KO hippocampus. Inhibition of PAK rescues morphological (spine density and morphology) and behavioral symptoms of fragile X syndrome in mice (Hayashi et al., 2007).

Dock4 is a risk gene for autism spectrum disorder (ASD) and other neuropsychiatric disorders. Dock4 encodes for a Rac1 guanine nucleotide exchange factor. Dock4 KO mice exhibited ASD-like behaviors, including elevated anxiety, abnormal isolation-induced pup vocalizations, impaired social novelty preference and perturbed object and spatial learning. Hippocampal neurons of KO mice show attenuation in excitatory synaptic transmission (in CA1), decreased spine density (in CA1 and DG), and synaptic content of AMPA and NMDA receptors (in whole hippocampus). Rac1 activity is reduced in the Dock4-deficient hippocampus, leading to the downregulation of protein synthesis and reduced expression of AMPA and NMDA receptor subunits. Injection of lentivirus expressing Rac1 into Dock4 KO mice hippocampal CA1 rescued excitatory synaptic transmission and plasticity impairments and corrected the impaired social deficits in these mice (Guo et al., 2019).

Rac1 has also been associated with major depressive disorder (MDD). A repressive chromatin state surrounding the Rac1 promoter and reduced Rac1 transcription in the NAc is found in subjects with MDD. In mice that underwent social defeat, a model of depression-like behavior, Rac1 mRNA level is downregulated. This reduction in Rac1 mRNA level is associated with a repressive chromatin state surrounding the proximal promoter of Rac. Reduction in Rac1 activity or its expression in the NAc of mice increases social defeat-induced social avoidance and anhedonia. The observations in the study indicate that the chronic social defeat stress-induced decrease in Rac1 expression results in concurrent stubby spine formation and cofilin localization within these spines. Expression in the NAc of constitutively active Rac1 after chronic social defeat stress reversed the induction of stubby spines and depression-related behaviors in mice (Golden et al., 2013).

In addition to psychiatric disorders, Rac1 is also associated with neurodegenerative brain disorders such as Alzheimer’s disease (AD). The pathological hallmarks of AD range from an extracellular accumulation of amyloid β plaques and neurofibrillary tangles to alterations in synaptic activity and memory loss (Small and Duff, 2008; Selkoe and Hardy, 2016; Femminella et al., 2018). Recently, Rac1 activity was demonstrated to be enhanced not only in AD patients but also in the hippocampus of APP/PS1 AD mice model and in a transgenic fly model of AD in comparison to the controls (Wu et al., 2019). This study further shows that Aβ42 oligomer application to HEK-293 leads to an increase in Rac1 activity. In addition, it is demonstrated that the impaired performance in APP/PS1 mice during the Morris water maze task could be rescued by the intragastric application of a Rac1 inhibitor, EHop-016. Furthermore, injection of a dominant-negative form of Rac1 into the hippocampus was able to inhibit the accelerated memory decay in the mutant mice. Lastly, LTP fast decay in the AD model was rescued as well by the application of the Rac1 inhibitor EHop-016 (Wu et al., 2019). Rac1 is also involved in another type of AD mice model, the 3xTg-AD. It is shown that Rac1 activity is increased in 6-week-old 3xTg-AD mice (Borin et al., 2018). Moreover, in primary cortical neurons, Rac1 or constitutively active mutant forms of Rac1 constructs led to the creation of pathogenic Aβ fragments and the translocation of SET from the nucleus to the cytoplasm, which also resulted in increased phosphorylation of tau. The authors also show that the levels of Rac1 in AD patients and 7-month-old 3xTg-AD mouse appeared to be significantly lower, which coincides with the decline of cognitive function in the mouse model. Additional to the decline of cognitive function and abnormal level of Rac1, dendritic spines were shown to be significantly reduced in the 7-month-old mouse model. The authors suggest a possible dual role of Rac1 according to the different stages of the pathology. Taken together, the results suggest that alteration in Rac1 activity is involved in AD-related memory abnormalities and the pathological changes that occur in the AD brain. Moreover, it appears that the reversal of the abnormal activity of Rac1 might restore some cognitive functions and morphological alterations, making this small GTPase an interesting target for study as a molecular marker and a possible target for pharmacological treatment.

Rac1 activity is also associated with Huntington’s disease (HD) that usually causes movement and cognitive and psychiatric disorders with an extensive spectrum of symptoms and signs (Walker, 2007). Rac1 activity levels are increased in the striatum of a 1.5-month-old mouse of HD Q140/Q140 mice (Knockin mouse with a chimeric mouse/human exon 1 containing 140 CAG repeats inserted in the murine huntingtin gene) but reduced in 4.5 months old mouse compared to controls. Huntingtin was found to associate with p85α subunit of the PI 3-kinase, actinin-2 and preferentially with active Rac1 (Tousley et al., 2019). Puigdellívol et al. (2015) detected a decrease in Rac1 activity in HD-mutant mice (Q7/Q111) (containing alleles with 7 CAG repeats and with targeted insertion of 109 CAG repeats that extends the glutamine segment in huntingtin to 111 residues) in the cortex. Kalirin-7, an activator of Rac1, was found to be significantly reduced in the cortex of these mice. The Hdh^Q7/Q111^ mouse model presented smaller spine density in the motor cortex but not striatum. In addition, the mice were impaired in the ability to learn new motor skills (Puigdellívol et al., 2015).

The above examples show that a dysfunctional Rac1 activity and level in the brain leads to neuronal morphological abnormalities, including these of dendritic spines. Moreover, the results indicate that such dysfunctions are involved with behavioral abnormalities associated with mental disorders. These observations give incentive to further explore the possibility that intervention in Rac1 level of activity can rescue such abnormalities in human brain structure and behavior.

## Conclusion

Rac GTPase regulates several signaling pathways in neurons including pathways that control actin cytoskeleton dynamics and structure. Such changes in actin dynamics and structure mediate spines morphogenesis and density and synaptic transmission in spine. As described above, several studies have shown that Rac-induced alterations in spine morphology correlate with the effects of Rac on memory formation.

The following model can be deduced from the aforementioned observations. It is possible that synaptic activation, for example during learning or memory extinction, leads to changes in Rac activity that in turn affects the actin cytoskeleton. Changes in actin cytoskeleton alter neuronal morphology. Such changes in the actin cytoskeleton are preserved over time [see for possible mechanisms of preserving such molecular and morphological changes over time in Basu and Lamprecht (2018)]. These modifications in neuronal morphology alter the responsiveness of the neurons to incoming sensory input, such that subjecting the animal to the sensory stimulus that participated in learning and led to the formation of memory (i.e. the conditioned stimulus) will lead to an activation of the memory trace neuronal circuit in the brain and to memory retrieval.

Rac1 GTPase is also important for postlearning functions such as erasure and forgetting. Thus, Rac1 activity can be used to modulate neuronal morphology after learning to control memory. Therefore, a balanced and controlled Rac activity following memory consolidation will determine whether the memory will be preserved or will deteriorate.

The above studies also show that the activity of Rac is essential for normal brain functions. Indeed, spine abnormal morphology and densities are observed in brain disorders where Rac and its regulators can be found in abnormal levels or dysregulated in terms of activity. It would be important to examine the possibility that controlling Rac activity can rescue memory-related brain disorders.

## Author Contributions

All authors listed have made a substantial, direct and intellectual contribution to the work, and approved it for publication.

## Conflict of Interest

The authors declare that the research was conducted in the absence of any commercial or financial relationships that could be construed as a potential conflict of interest.
